# Seasonality in daily movement patterns of mandrills revealed by combining direct tracking and camera traps

**DOI:** 10.1093/jmammal/gyab141

**Published:** 2021-11-25

**Authors:** Shun Hongo, Yoshihiro Nakashima, Etienne François Akomo-Okoue, Fred Loïque Mindonga-Nguelet

**Affiliations:** 1 The Center for African Area Studies, Kyoto University, 46, Yoshida Shimoadachi-cho, Sakyo-ku, Kyoto, Kyoto, Japan; 2 Department of Forest Science and Resources, Nihon University College of Bioresource Science, 1866, Kameino, Fujisawa, Kanagawa, Japan; 3 Institut de Recherche en Écologie Tropicale, Centre National de la Recherche Scientifique et Technologique (IRET–CENAREST), B.P. 13354, Libreville, Estuaire, Gabon

**Keywords:** day range, direct observation, GAMM, Moukalaba-Doudou, movement rate, multiple-method approach, primate, rainforest, travel speed

## Abstract

Movement is a fundamental characteristic of animals, but challenging to measure noninvasively. Noninvasive methods for measuring travel have different weaknesses, so multiple techniques need to be applied multiple techniques for reliable inferences. We used two methods, direct tracking and camera trapping, to examine the variation in time and seasonal differences in movement rates of mandrills (*Mandrillus sphinx*), an elusive primate that lives in large groups in central Africa. In a 400-km^2^ rainforest area in Moukalaba-Doudou National Park, Gabon, we tracked unidentified groups 46 times from 2009 to 2013. We systematically placed 157 terrestrial camera traps in the same area from 2012 to 2014 and recorded groups 309 times. Generalized additive mixed models (GAMMs) of the tracking data indicated that the group travel speed varied with time and season. In the fruiting season, the movement rate fluctuated with time in a bimodal pattern, whereas in the nonfruiting season, it increased monotonously with time. The predicted day range was longer in the fruiting season (6.98 km) than in the nonfruiting season (6.06 km). These seasonal differences suggest responses to changes in food resources and temperature. Camera-trap detection rates showed similar temporal and seasonal patterns to the tracking data, allowing us to generalize our findings to the population level. Moreover, cameras never detected mandrills at night, and we observed that they slept high in trees and hardly moved until the next morning, all suggesting their strict avoidance of nighttime movement. This study demonstrated the significance of the multiple-method approach in drawing robust conclusions on temporal patterns of animal movement.

## Abstract (es)

Le mouvement est une caractéristique fondamentale des animaux, mais il est difficile de le mesurer de manière non invasive. Les méthodes non invasives de mesure des déplacements présentent des faiblesses différentes, de sorte que nous devons appliquer plusieurs techniques à un même sujet pour obtenir des déductions fiables. Nous avons utilisé deux méthodes, le suivi direct et le piégeage photographique, pour examiner les variations temporelles et la différence saisonnière des taux de déplacement des mandrills (*Mandrillus sphinx*), un primate insaisissable vivant en grands groupes. Dans une zone de forêt tropicale humide de 400 km^2^ du parc national de Moukalaba-Doudou au Gabon, nous avons suivi des groupes non identifiés 46 fois entre 2009 et 2013. Dans la même zone, nous avons systématiquement placé 157 pièges photographiques terrestres de 2012 à 2014 et enregistré des groupes 309 fois. Les modèles mixtes additifs généralisés (GAMM) des données de suivi ont indiqué que la vitesse de déplacement des groupes variait en fonction du temps et des saison. Pendant la saison de fructification, la vitesse de déplacement fluctuait dans le temps selon un schéma bimodal, tandis que pendant la saison de non-fructification, elle augmentait de façon monotone avec le temps. Les distances journalières prédites étaient plus longues pendant la saison de fructification (6,98 km) que pendant la saison de non-fructification (6,06 km). Ces différences saisonnières suggèrent des réponses aux changements de ressources alimentaires et de température. Les taux de détection des pièges photographiques ont montré des tendances temporelles et saisonnières similaires à celles des données de suivi, ce qui nous permet de généraliser nos conclusions au niveau de la population. De plus, les caméras n’ont jamais détecté les mandrills la nuit, et nous avons observé qu’ils dormaient haut dans les arbres et ne bougeaient pratiquement pas jusqu’au lendemain matin, ce qui suggère qu’ils évitent strictement de se déplacer la nuit. Cette étude a démontré l’importance de l’approche à méthodes multiples pour tirer des conclusions solides sur les modes temporels de déplacement des animaux.

Movement is a fundamental characteristic of animals with each species adjusting the movement patterns differently with time and season to meet various ecological needs (Nathan et al. 2008) such as foraging (Grotta-Neto et al. 2019; Hanya et al. 2020); mating and reproduction (Mizumoto et al. 2020); thermoregulation (Spitz et al. 2018; Zollner et al. 2020); and avoidance of predators (Pokallus and Pauli 2016; Picardi et al. 2019), parasites (Kärvemo et al. 2020), and human disturbance (Tucker et al. 2018; Hertel et al. 2021). Researchers have used different methods to study animal movement. Among these, telemetry is an increasingly popular option that involves the attachment of Global Positioning System (GPS) devices, which allows collecting positional data of focal animals with high temporal and spatial resolution over several years (Hussey et al. 2015; Kays et al. 2015). This technology, however, requires capture and immobilization of the animal with additional energy expenditure while carrying the device, which raises ethical issues (McIntyre 2015) and confounds movement behavior (Dechen Quinn et al. 2012).

In cases where telemetry devices are not preferable or feasible, including studies on protected populations or elusive species, researchers have to employ other methods to collect the movement data. Direct tracking of individual animals or groups of animals is a traditional method to estimate the movement rate (distance traveled per unit time) and day range (daily travel distance) of large mammals (Matsumoto-Oda 2002; Kolowski et al. 2007). It allows detailed movement and behavioral recording without the need for capture (Peterson and Weckerly 2018; Saito and Idani 2020). This method, however, demands a high amount of time and labor, especially for species that require habituation to observe their natural movements. Consequently, records are often fragmented and collected from only a small number of focal individuals or groups, making it difficult to generalize findings to the population level. Moreover, tracking is highly challenging at night. These weaknesses are particularly evident when studying animals living in closed habitats with long day ranges.

Alternatively, motion-triggered trail cameras, often called camera traps, have the potential to indirectly elicit information on the temporal patterns of animal movement from image records. Camera trapping is now a standard method in studying animal behavior and ecology (O’Connell et al. 2011; Burton et al. 2015; Caravaggi et al. 2017). In addition to minimal impact on animal behavior, this technique allows 24-hour monitoring of terrestrial animals, albeit over a small area, and enables population-level inference by random or systematic camera placement.

Rowcliffe et al. (2016) proposed an approach to estimate the average movement rate and day range of terrestrial animals using camera traps. This method requires fine-scale measurements of recorded movement, which represents considerable labor in the field (Palencia et al. 2019). Instead, the relative variation in movement rates over time can be inferred based on the detection rate (the number of camera-trap records per unit time) of focal animal individuals or groups. Rowcliffe et al. (2014) formulated four determinants of detection rate at a given time: animal density, camera detection area size, movement rate, and active proportion (number of individuals or groups in the population on the move). It is reasonable to assume a constant animal density over the daily cycle when cameras are placed randomly or systematically. In addition, the size difference in the detection area between day and night should not be problematic for noncathemeral species. Therefore, if the constancy of active proportion can be confirmed by another method, time variation in detection rates directly translates into the temporal pattern of movement rates.

Each method used in animal movement research has its strengths and weaknesses. Therefore, it is essential to apply different approaches to the same question for reliable inferences. Comparison and integration of results from multiple methods, termed triangulation by Munafò and Davey Smith (2018), is crucial for verifying and generalizing the scientific findings (Nuñez et al. 2019). Studies on animal movement, however, have rarely applied multiple independent field methods to the same population (but see Fedigan et al. 1988; Thompson et al. 2018).

The present study examines seasonality in the temporal patterns of group movement rates of mandrills (*Mandrillus sphinx*) by integrating data from direct tracking and camera trapping. This endangered primate, classified as Vulnerable on the IUCN Red List (Abernethy and Maisels 2019), inhabits rainforests in central Africa and has an omnivorous diet with a preference for fruits (Abernethy and White 2013). Mandrills typically live in female-biased groups of 300 to >800 individuals (Abernethy et al. 2002; Hongo 2014) with a vast home range of ~50 km^2^ (White et al. 2010). Large groups travel mainly on the ground during the day and sleep in trees at night (Hoshino et al. 1984). Given that several studies have reported seasonal changes in their diet (Hoshino 1985; Nsi Akoue et al. 2017), habitat use (Hongo et al. 2018), and reproduction (Setchell et al. 2002; Setchell and Wickings 2004; Hongo et al. 2016), we expected that the movement rate patterns would also vary seasonally. Nevertheless, our knowledge of mandrill movement patterns is quite limited because the dense rainforest vegetation, large group size, and large home range size prevent continuous tracking. To date, only a tracking (Hoshino 1985) and telemetry (Brockmeyer et al. 2015) study have estimated day ranges of small groups (95–120 individuals) and reported that the groups traveled more during the fruiting season.

We hypothesized that large mandrill groups would change their temporal movement patterns in response to the seasonal environments of African rainforests. We conducted three lines of data collection and analysis. First, we employed a direct tracking method on several unidentified groups for as long as possible to record their movement patterns. The constancy of the active proportion and the seasonal difference in the group movement rate patterns were then examined. We predicted that mandrill groups seasonally change their temporal movement patterns according to fruit availability. Second, we used camera traps systematically installed in the study area to record the same mandrill groups. The seasonal difference in temporal patterns of the detection rate was examined employing the method of Rowcliffe et al. (2014). We predicted that daily patterns of the detection rates also vary with the seasonal change in fruit availability. Lastly, we compared the results from the two methods by examining the similarity in the shape of temporal patterns between the direct-tracking movement rate and the camera-trap detection rate. We predicted that if the two methods accurately capture the seasonality in daily movement behaviors, the resulting patterns from the two data sets will be similar to each other.

## Materials and Methods

This study complied with American Society of Mammalogist guidelines (Sikes et al. 2016) and the Gabonese Republic laws. We conducted fieldwork with approval from the Centre National de la Recherche Scientifique et Technologique (CENAREST, N° AR0031/11/MENESRSIC/CENAREST/CG/CST/CSAR) and the Agence Nationale des Parcs Nationaux (ANPN, N° 000017/PR/ANPN/SE/CS/AEPN, N° 000022/PR/ANPN/SE/CS/AEPN).

### Study area and study population

**Fig. 1.**
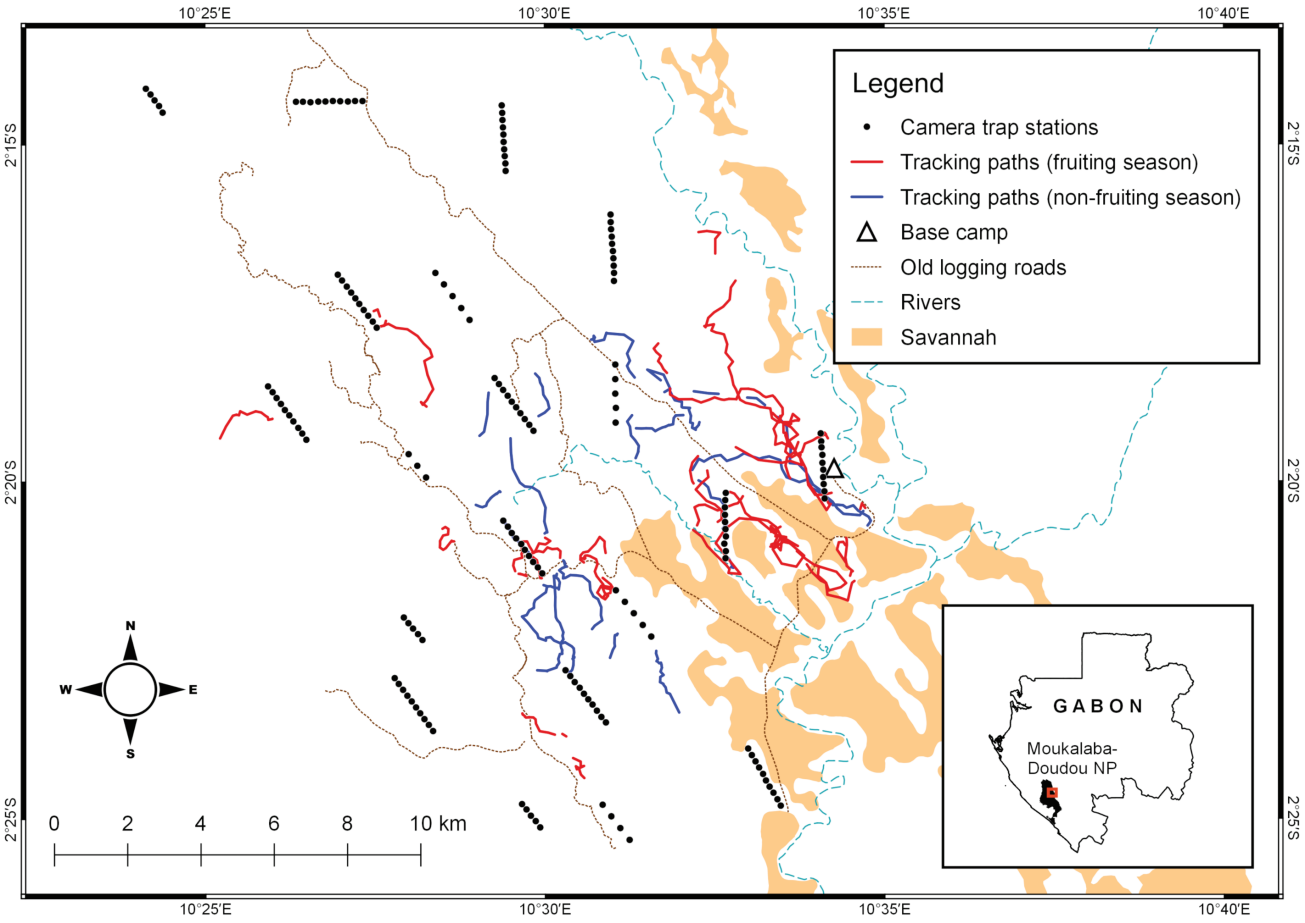
—Map of the study area in Moukalaba-Doudou National Park, Gabon, depicting tracking paths of mandrill groups (2009–2013) and camera-trap stations (2012–2014).

We conducted the study within 400 km^2^ in the eastern part of Moukalaba-Doudou National Park, Gabon. Our base camp was located at S2°19′ and E10°34′. This area, close to the southern limit of mandrills’ geographic range (Abernethy and Maisels 2019), included savannah and different vegetation types of forests (Fig. 1). We have been studying mandrills in this area since 2008 and successfully counted three groups of 169, 350, and 442 individuals (Hongo 2014), but no groups have yet been habituated.

The study area exhibits seasonality in the rainfall and temperature. With the annual rainfall of approximately 1,700 mm, monthly amounts vary seasonally. Typically, monthly rainfall exceeds 100 mm from October to April, whereas it drops below 100 mm from May to September. The rainy periods are, however, interrupted irregularly by low-rainfall months (Takenoshita et al. 2008). During the entire study period (55 months from August 2009 to February 2014), there were five months with the rainfall <100 mm between October and May (January and December in 2011; January, March, and April in 2012). Monthly maximum and minimum temperatures ranged from 27 to 34°C and 19 to 24°C, respectively, and fluctuated with the season (about 5°C higher in October–May than in June–August).

Fruit production and mandrill diets also show regular and seasonal fluctuations in the study area. Based on a 2-year transect survey in 2012–2013, the mean densities of fallen fruit clusters were highest (30–40 clusters/km) in December–February, decreased substantially in March–August (5–10 clusters/km), and increased again in September–November (15–25 clusters/km) (Hongo et al. 2018). Mandrills adjust their diets to the fruiting phenology. They feed on more pulp of fruits (including *Cola* spp., *Gambeya africana*, and *Santiria trimera*) from September to February, whereas they eat more woody tissue and increase dietary diversity from March to August (Hongo et al. 2018). From these findings, we defined September to February as the fruiting season and March to August as the nonfruiting season.

### Direct tracking

Over 25 months between August 2009 and September 2013, SH conducted foot patrols with well-trained field assistants to search for mandrill groups throughout the study area. We organized one to three search teams of two or three people on each day of the patrol. Upon finding a group, we tracked it from about 50 m behind to avoid disturbing its movement. Visibility in the forest was generally around 30 m, so we tracked the groups relying on the females’ long-distance calls (Kudo 1987). We continued the tracking sessions until we lost the group or observed mandrills beginning to sleep in the trees. We tracked the groups only between 06:00 and 18:00 because nighttime fieldwork was not permitted by ANPN for safety reasons.

During the tracking sessions, we recorded the coordinates and elevation about every 15 min using a GPSmap 60csx/62sportable device (Garmin International, Inc., Olathe, Kansas). When lagging more than 100 m behind the group, we stopped the recording until we caught up with the group again.

### Camera trapping

YN, SH, EFA-O, and FLM-N conducted a camera-trap survey continuously from January 2012 to February 2014. This survey was initially designed to estimate the population density of forest duikers (Nakashima et al. 2020). We used the Bushnell Trophy Cam (model numbers 119435 and 119436, Overland Park, Kansas) with a maximum detection distance of approximately 10 m and a 50° field of view. Cameras recorded a 30-s video file in response to an animal passage with a 30-s minimum interval before a new recording could be triggered.

We installed cameras at 157 stations with three to 10 stations set at regular intervals of 200 or 400 m along 20 line transects of 1–2 km (Fig. 1b). We did not intentionally select animal trails or particular vegetation types for camera stations, nor did we use bait or lures. We strapped each camera to a tree 10 m away from the transect and adjusted it to be parallel to the ground at the height of 30 cm. We then cleared the undergrowth in front of cameras to ensure detection of animal passages. We checked the cameras on a monthly basis.

### Data analysis

SH and YN performed statistical analyses using R version 4.0.3 (R Core Team 2020) and RStudio version 1.4.1106 (RStudio Team 2021). All statistical tests were two-sided, and we considered *P*-values <0.05 as statistically significant. All the data analyzed and the R code used in the study are accessible in a Dryad data repository (Hongo et al. 2021).

#### Data analysis 1: tracking data.—

We used the tracking data to examine active proportion and temporal movement rate patterns. Tracking sessions that lasted <30 min were discarded from the analysis because the presence of observers probably had affected the group movement. From the remaining 46 sessions, we calculated the movement distances and time durations from the GPS tracking points (*N* = 690).

We estimated the measurement error distribution of GPS positioning in our study area by leaving the GPS device in the forest for 225 min and recording the same point coordinates every minute. The GPS measurement error distribution was constructed using the distances between consecutive coordinates in a generalized linear model following a gamma distribution. The estimated mean measurement error was 8.8 m, and its 95% prediction interval was 1.4–24.5 m.

To examine the active proportion, we categorized the 690 data points as either a move or pause. Based on field observations and the GPS measurement error distribution estimated, we judged that the groups paused when the movement rate was <25 m per 15 min.

To model the effects of time and season on the group movement rate, we constructed generalized additive mixed models (GAMMs) using the R package “gamm4” version 0.2-6 (Wood and Scheipl 2020). We assumed the response variable (movement distance in meters) followed a gamma distribution, and used the log link function. The linear predictor of the full model contained the following components: an offset term of time duration in minutes, three fixed effects (a smooth term of time of day, a parametric term of season [fruiting, nonfruiting] and the interaction between time of day and season effects), and a random smooth effect (the effect assuming that the temporal patterns of movement rate are randomly different between the tracking sessions). We tested for the fixed interaction effect and the random smooth effect with likelihood ratio tests using the “anova” function. We also performed model validation for the full model and the optimal model (the model including only significant effects) by graphically checking the residual plots and the normal Q–Q plots (Zuur et al. 2009). Finally, we estimated mean day ranges from 06:00 to 17:30 based on the optimal model.

#### Data analysis 2: camera-trap data.—

Our terrestrial camera traps functioned for a total of 64,854 camera days (mean number of working cameras per day = 84.4 ± *SD* 22.8, range: 7–128). We used only mandrill group records of videos showing at least two or more reproductive females or immatures for the analysis. We discarded detections of solitary males and those whose social composition was unclear. In cases where a group triggered the same camera repeatedly at intervals of <10 min, we considered these consecutive records as a single detection of a group and used only the time of the initial trigger (Rowcliffe et al. 2014). As a result, we obtained 309 detections along 15 transects.

We modeled the temporal variation in the detection rates as Von Mises kernel probability density functions using the “fitact” function in the R package “activity” version 1.3 (Rowcliffe 2019). Modeling the data of the fruiting and nonfruiting seasons separately, we tested for the seasonal difference in the shape of temporal patterns using a randomization test of the “compareCkern” function in the “activity” package.

#### Data analysis 3: comparison between direct tracking and camera trapping.—

To examine the similarity in the shapes of temporal patterns obtained by the two methods, we remodeled the temporal variation in the camera-trap detection rate while correcting the movement rate effect. We first generated the predicted values of the mean movement rate at each camera-trap detection time from the optimal model in the direct tracking analysis. We then weighted the camera-trap detection probability with the inverse of the predicted mean movement rate using the “wt” argument in the “fitact” function. We expected that if the original patterns of the detection rate are functions of movement rates only, the resulting weighted patterns would be flat throughout the daytime.

## Results

### Tracking data.—

In the 46 tracking sessions, we tracked mandrill groups for 2.6 ± 2.0 ($\overset{\pm }{x}$ ± *SD*) km during a mean of 3.8 ±2.3 ($\overset{\pm }{x}$ ± *SD*) hours (Table 1). We never observed mandrill groups traveling through the savannah (Fig. 1).

**Table 1.** Descriptive statistics on direct tracking (2009–2013) and camera trapping (2012–2014) of mandrill groups in Moukalaba-Doudou National Park, Gabon.

	Season		Total
	Fruiting (September–February)	Nonfruiting (March–August)	
Direct tracking			
No. tracking sessions	30	16	46
Tracking distance (km)^a^	2.4 ± 2.2 (0.1–10.4)	2.9 ± 1.9 (0.1–5.9)	118.2
Time duration (h)^a^	3.2 ± 2.1 (0.5–8.3)	4.8 ± 2.4 (0.6–8.7)	172.7
Movement rate (km/h)^a^	0.7 ± 0.3 (0.2–1.5)	0.6 ± 0.3 (0.2–1.0)	–
Camera trapping			
No. detections	199	110	309
Detection time (earliest–latest)	06:39–18:17	07:00–18:04	–

^a^These values are presented as “mean ± *SD* (min.–max.)” for each season.

**Fig. 2.**
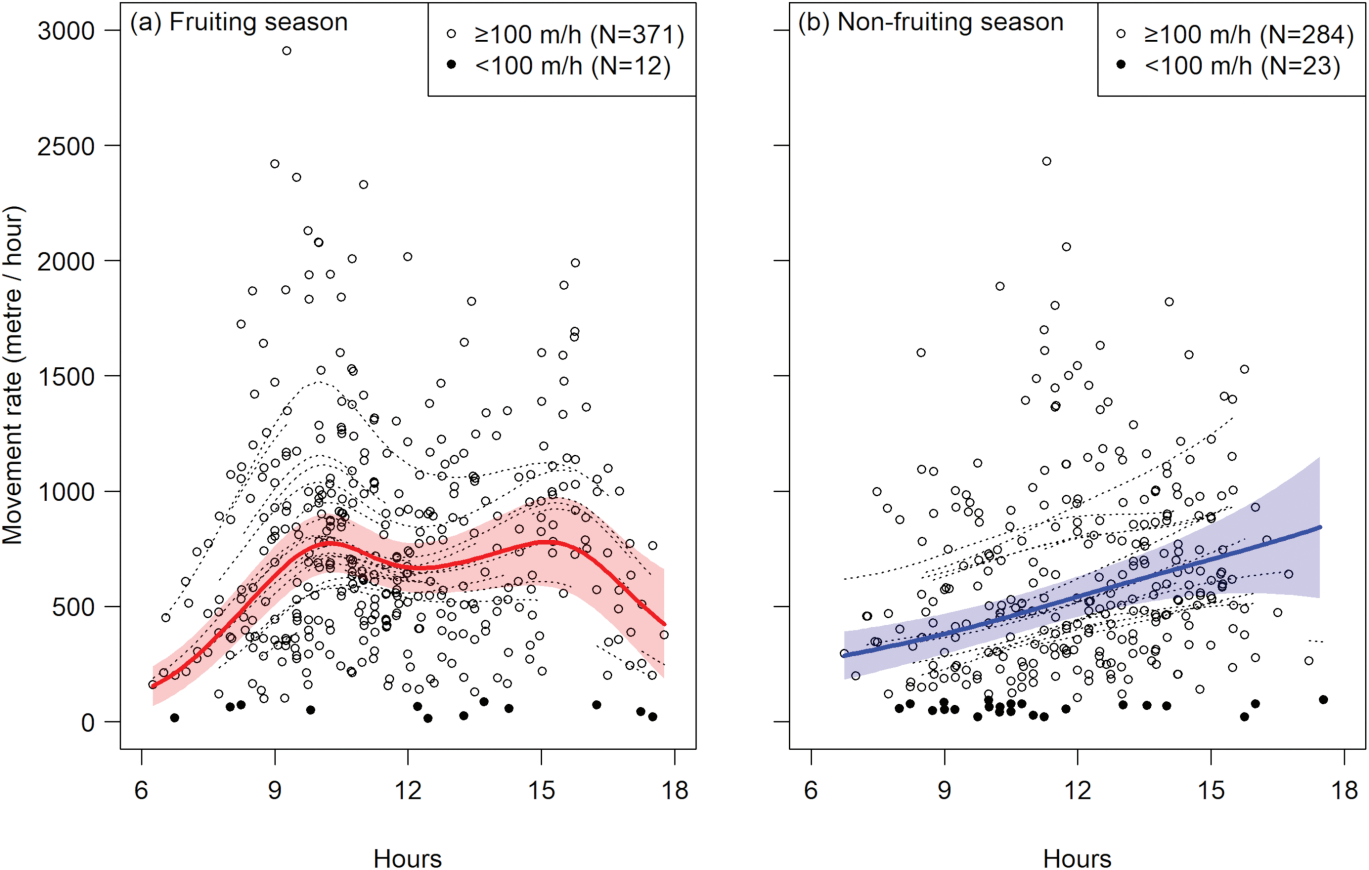
—Temporal variations in the movement rate of mandrill groups during (a) the fruiting season and (b) the nonfruiting season in Moukalaba-Doudou National Park, Gabon. White circles show data points at which groups were moving, and filled circles represent those for temporal pauses. Bold lines indicate the predicted general trend based on the optimal model, and shaded areas represent the 95% *CI*. Dotted curves show the random smooth effect—predictions for each tracking session.

Group active proportion was high throughout the daytime with tracking points considered to be a group pause (i.e., movement rate of <100 m/h) accounting for only 3.1% in the fruiting season and 7.5% in the nonfruiting season (Fig. 2). We observed group members sleeping >20 m high in the canopy during four sessions, of which all were after 17:44. In the early morning of the following days, we found the groups in almost the same places.

As a result of likelihood ratio tests, both the fixed interaction effect (Δ*D*_1_ = 8.70, *P* = 0.013) and the random smooth effect (Δ*D*_1_ = 9.74, *P* = 0.0077) of the movement rate GAMM were statistically significant. This suggests that temporal patterns of the movement rate variations randomly differed between sessions and there was a general difference between the two seasons. According to the optimal model (Table 2), the movement rate variation showed a bimodal shape in the fruiting season, with peaks at 10:10 and 15:10 and a slight trough at 12:20 (Fig. 2a). The nonfruiting season trend, in contrast, was a monotonous increase with time (Fig. 2b). The curve in the nonfruiting season did not turn downward until sunset, probably because of the small sample size near dusk. Mean day ranges predicted by the optimal model were longer in the fruiting season (6.98 km) than in the nonfruiting season (6.06 km).

**Table 2.** Parameter estimates of the optimal model for the movement rate of mandrill groups in Moukalaba-Doudou National Park, Gabon.

Model structure	α + *f*_1_(*T*) + β _s_ × *f*_2_(*T*) + *r*_sm_
Fixed effect	
Parametric term [estimate (95% *CI*)]	
Intercept [α]	6.50 (6.37 to 6.64)
Season (nonfruit) [β _s_]	−0.26 (−0.47 to −0.05)
Smooth term [edf]	
Time of day × Season (fruit) [*f*_1_(*T*)]	4.89
Time of day × Season (nonfruit) [*f*_2_(*T*)]	1.42
Random effect (standard deviation)	
Random smooth for time of day [*r*_sm_]	1.61

### Camera-trap data.—

**Fig. 3.**
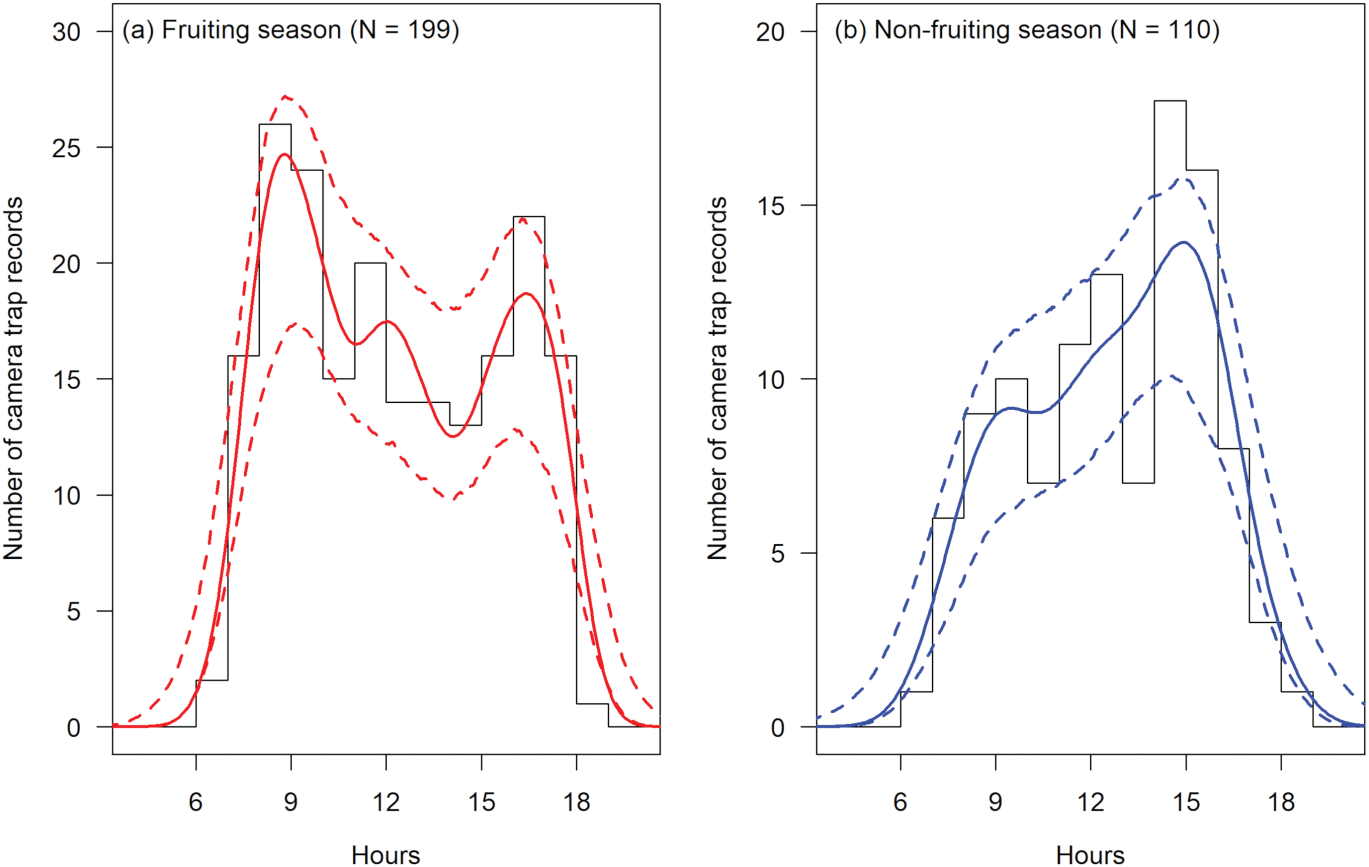
—Temporal variations in the camera-trap detection rate of mandrill groups during (a) the fruiting season and (b) the nonfruiting season in Moukalaba-Doudou National Park, Gabon. Histograms represent the data frequency. Bold and dotted curves show the estimated kernel densities and their 95% *CI*, respectively.

Our terrestrial camera traps detected mandrill groups 309 times between 06:39 and 18:17 (Table 1). The shapes of temporal variation in the detection rate were statistically different between the fruiting and nonfruiting seasons (randomization test: observed overlap index = 0.829, mean null overlap index = 0.904, *SD* of the null distribution = 0.030, *P* = 0.015). In the fruiting season, the temporal variation had a bimodal shape with peaks at 08:50 and 16:20 and a trough at 14:10 (Fig. 3a). In the nonfruiting season, the shape was unimodal with a peak at 14:50 (Fig. 3b).

### Comparison between direct tracking and camera trapping.—

**Fig. 4.**
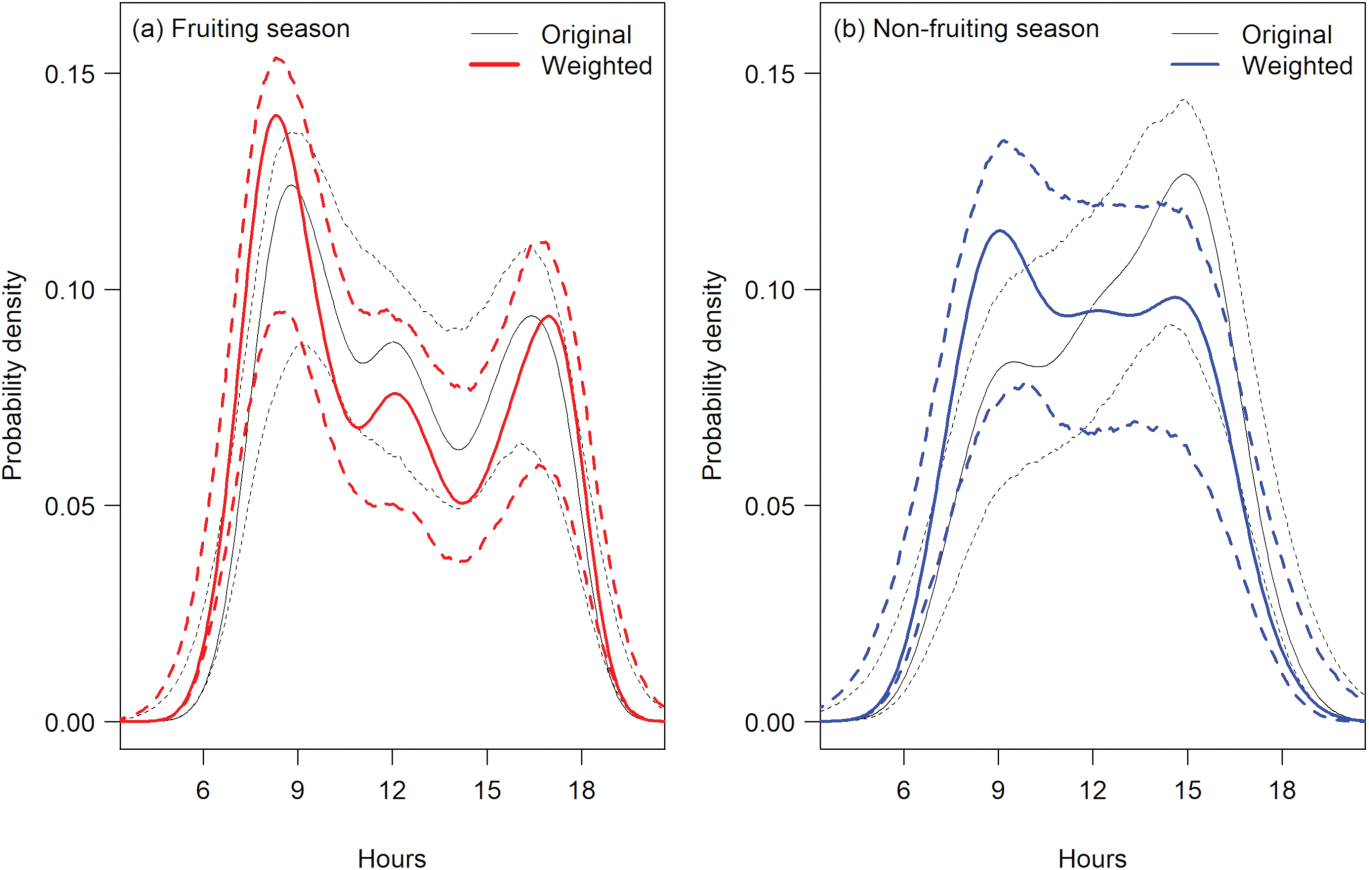
—Corrected temporal variations in camera-trap detection rates of mandrill groups during (a) the fruiting season and (b) the nonfruiting season in Moukalaba-Doudou National Park, Gabon. The weighted patterns corrected for the movement rate effect (bold lines) are compared with the original patterns (fine lines, same as in Fig. 3). Solid and dotted curves show the estimated functions and the 95% *CI*, respectively.

After correcting for the movement rate effect, temporal variation patterns of the camera-trap detections changed their shapes. The shape for the fruiting season model altered only slightly: The two peaks slightly shifted towards twilight with the morning peak becoming more pronounced (Fig. 4a). For the nonfruiting season model, the afternoon peak became lower after the correction, leading to a flatter pattern throughout the daytime (Fig. 4b).

## Discussion

Animal movement is a fascinating, but difficult behavior to measure noninvasively and different methods have strengths and weaknesses. We examined the seasonality in movement rate patterns of mandrill groups using two methods—direct tracking and camera trapping. Direct tracking revealed that the groups continuously moved throughout the daytime, without long pauses, and avoided the savannah. Group movement rate varied with time and season. In the fruiting season, the group day range was longer, and the temporal movement rate formed a bimodal pattern. In the nonfruiting season, the day range was shorter, and the movement rate increased monotonously with time during the day. Terrestrial camera traps detected the groups only in the daytime. The detection rate generally replicated the movement rate patterns. Correcting for the movement rate effect, the pattern of daytime detection rate flattened in the nonfruiting season, but a flattening did not occur in the fruiting season.

By integrating the two methods, we reliably revealed the seasonality in the movement rate patterns, as predicted. Our direct tracking showed a constantly high active proportion of the groups through the daytime (Fig. 2), suggesting that the camera-trap detection rate was a function of the movement rate alone (Rowcliffe et al. 2014). The estimated patterns of the detection rates confirmed this suggestion. Similarly for both methods, the temporal variation formed a bimodal pattern in the fruiting season and a monotonous increase in the nonfruiting season (Figs. 2 and 3). Our tracking data had a small sample size and most tracking sessions did not last throughout the daytime (Table 1). Patterns derived from this fragmented tracking data, nevertheless, have been replicated by our systematic camera trapping. This allows us to generalize our findings to the study population.

Why do mandrills change the group movement patterns between seasons? We hypothesize that they respond to seasonal changes in food resources and temperature. Slower general movement rates and a shorter day range in the nonfruiting season may be due to dietary shifts. In the fruiting season, mandrills mainly eat fresh fruits, which are clumped in trees. In the nonfruiting season, they feed on dispersed foods such as buried seeds and woody tissue in the forest litter (Hoshino 1985; Hongo et al. 2018). Large mandrill groups in our study area may need to move slower to forage for the dispersed foods during the season when the preferred foods are scarce, as observed in smaller groups in other sites (Hoshino 1985; Brockmeyer et al. 2015) and other primates (Hemingway and Bynum 2005; Tsuji 2010; Reyna-Hurtado et al. 2018; Green et al. 2020).

The bimodal movement pattern observed in the fruiting season may be the result of thermoregulation. The ambient temperature increases during the fruiting season, often exceeding 35°C around noon (Takenoshita et al. 2008). The observed decrease in the movement rate during this season suggests that many individuals in the group relaxed their foraging activity in midday to avoid excessive body temperature increases. Behavioral thermoregulation is crucial for mammals to maintain homeostasis (Weiss and Laties 1961; Terrien et al. 2011). Reduced movement and activity during the hottest hours are reported in many diurnal mammals, including Cabrera voles (*Microtus cabrerae*) (Grácio et al. 2017), Verreaux’s sifakas (*Propithecus verreauxi*) (Erkert and Kappeler 2004), and moose (*Alces alces*) (Alston et al. 2020), as well as birds (Silva et al. 2015) and lizards (Foa and Bertolucci 2001).

Although the two methods exhibited similar temporal patterns in each season, our analytical comparison showed that the fruiting-season patterns were not exactly consistent between the methods (Fig. 4a). Part of this inconsistency may be because two study periods did not overlap exactly. Direct tracking was conducted between 2009 and 2013 and the camera-trap survey was carried out from 2012 to 2014. Group movement patterns were different from day to day (Figs. 1 and 2), so the noncoincident study periods may have led to the observed difference in the results. Also, the analytical methods were different (GAMM vs. kernel density function). These methodological differences may have generated the mismatch in patterns, as also reported in other studies applying multiple methods (Kamgaing et al. 2018; Steinbeiser et al. 2019; Wei et al. 2020). Notwithstanding these differences, we suggest there is a bimodal movement rate pattern in the fruiting season, but further investigation is needed to determine the intensity and time of the peaks.

An interesting secondary finding was that mandrill groups moved exclusively during the daytime. On four occasions, we observed mandrills beginning to sleep high in trees before 18:00, and the groups hardly moved until the next morning. Camera traps strongly supported these observations because they did not detect the groups between 18:30 and 06:30 (Table 1), regardless of the season. Several primate species living in much smaller groups sometimes travel on the ground at night (Hanya et al. 2018; Tagg et al. 2018), so it is surprising that the large mandrill groups totally avoided terrestrial movement at night, although they may be active in the trees (Mochida and Nishikawa 2014). Nocturnal ground movement is also reported in wild ring-tailed lemurs (*Lemur catta*)—a smaller-sized primate living in smaller groups than mandrills (LaFleur et al. 2014). Although mandrills are known prey of leopards (*Panthera pardus*) and central African pythons (*Python sebae*) (Henschel et al. 2011; Abernethy and White 2013), it is questionable whether this avoidance of nocturnal movement can be solely explained by predator avoidance. Future research is required to more closely study their nocturnal behavior to better understand predator–prey interactions and their influence on movements.

In conclusion, our multiple-method approach reliably revealed the seasonality in temporal movement patterns and the avoidance of nocturnal movement in mandrill groups. Direct tracking demonstrated that the groups constantly moved throughout the daytime. This enabled us to interpret the temporal variations in camera-trap detections as the movement rate variations. The absence of nighttime recordings during the extensive camera-trap survey reinforced the anecdotal observations of sedentary groups at night. The observed differences in the movement rate patterns during the two seasons suggested different responses to seasonal food resources and temperature. The strict avoidance of nocturnal movement implied predator avoidance, but further investigation is required to confirm this behavior. Because each wildlife research method has specific weaknesses, and researchers may have confirmation bias (Marsh and Hanlon 2007), applying multiple techniques to the same questions is essential for robust conclusions, particularly when studying elusive and endangered animals.
